# Beyond the Lesion: Neurorehabilitation as a Humanistic Enterprise

**DOI:** 10.3390/brainsci16101070

**Published:** 2026-10-06

**Authors:** Francisco Nieto-Escamez

**Affiliations:** 1 Department of Psychology, University of Almeria, Ctra. Sacramento S/N, 04120 Almeria, Spain; pnieto@ual.es; 2 Research Center for Well-Being and Social Inclusion (CIBIS), University of Almeria, Ctra. Sacramento S/N, 04120 Almeria, Spain

## 1. Rehabilitation as the Restoration of Human Experience

Rehabilitation in neurological disorders has long been framed as a technical endeavor: restoring gait, improving balance, enhancing cognitive performance, and optimizing daily functioning. Yet the studies gathered in this Special Issue reveal something deeper. They show that rehabilitation is not merely the recovery of function—it is the recovery of presence, agency, and continuity of self. When neurological injury disrupts the ability to act, remember, or navigate the world, what is threatened is not only the body but the personhood that animates it.

This Special Issue brings together diverse technological approaches—immersive virtual reality, mixed reality-based occupational therapy, advanced locomotion systems for severe stroke, balneotherapy for stress regulation, augmented and mixed reality for multiple sclerosis—and yet they converge on a shared insight: technology can support rehabilitation, but only when it remains anchored in the lived experience of the patient. The challenge is not simply to innovate, but to ensure that innovation does not eclipse humanity.

The first contribution of this Special Issue is conceptual. It suggests that rehabilitation should be understood not merely as the recovery of motor or cognitive abilities, but as the restitution of human experience itself. This perspective aligns with recent reflections in neurorehabilitation, which argue that recovery must be framed in terms of participation, identity, and lived experience—not just impairment metrics. An overreliance on population-level data risks sidelining the lived experiences and autonomous living goals that matter most to individual patients [1].

Immersive VR intervention for people with dementia exemplifies this shift. Latorre et al. (Contribution 1) showed that nature-based VR scenarios reduced heart rate over time and were rated positively by all participants. Yet the deeper significance lies beyond physiological modulation. These immersive environments create a protected experiential space where individuals can reconnect with sensations, memories, and landscapes that evoke meaning. Technology in neurorehabilitation is most valuable when it preserves and enhances the humanistic dimensions of care. The risk that technology-focused strategies overshadow individual needs is successfully avoided here by prioritizing experiential safety and emotional connection over technical performance metrics [2].

The locomotion studies in severe stroke, such as the pilot randomized controlled trial by Naidu et al. (Contribution 2), reinforce this idea. Even when 12 training sessions did not yield group-level clinically meaningful gains in walking speed, the attempt to restore mobility reflects a deeper aspiration: the return to spatial agency, to inhabiting one’s surroundings with autonomy. Rehabilitation, in this sense, is not only about gait parameters—it is about reclaiming the ability to move through the world.

Rapolienė et al. (Contribution 3) demonstrated that balneotherapy significantly reduced distress intensity and salivary cortisol levels, with winter interventions showing greater efficacy. Their findings illustrate that physiological regulation is inseparable from emotional safety. Meaningful outcomes in neurorehabilitation emerge when interventions support both biological adaptation and experiential well-being [3]. Emotional safety is not an accessory to rehabilitation; it is a therapeutic substrate.

Franzò et al. (Contribution 4) developed a mixed-reality occupational therapy protocol for ataxic patients, and participants expressed trust in the system and confidence in its rehabilitative potential. Meaningful activity—reaching, grasping, interacting with holographic guides—is not merely functional training. It is a way to re-engage with identity, competence, and agency—a process that lies at the heart of preserving the human core of rehabilitation when technology is used authentically [2].

Finally, the systematic review by Fernández-Cañas et al. (Contribution 5) on augmented and mixed-reality interventions in multiple sclerosis shows how these technologies can deliver engaging, multisensory, and task-specific therapeutic experiences while respecting patient variability and vulnerability. Intelligent technologies matter most when they enhance participation, meaning, and ecological validity [3].

Across these studies—whether through immersive environments that evoke meaning, mixed-reality protocols that restore agency, or technologies that respect vulnerability—rehabilitation emerges as a profoundly humanistic endeavor: a process of enabling people to re-engage with the world in ways that feel authentic, safe, and meaningful. It is not only the restoration of function; it is the restoration of experience.

## 2. The Dialectic of Technology: Promise and Peril

The second contribution of this Special Issue is dialectical. Technology in neurorehabilitation is not neutral. It carries a double tension: it can empower, but it can also constrain; it can humanize, but it can also dehumanize [2].

The promise is clear. VR can motivate and engage. Robotics can support movement that would otherwise be impossible. Mixed reality can scaffold complex tasks. Sensors can detect risk and prevent harm. These tools expand what clinicians can do and what patients can attempt.

But the peril is equally real. Technology can become prescriptive rather than supportive. It can impose rigid thresholds that fail to respect variability. It can overwhelm with sensory load. It can reduce the patient to a data point or a behavioral pattern. It can inadvertently shift the therapeutic relationship from a human encounter to a technical transaction. Consider, for instance, a VR system whose rigid design fails to accommodate the cognitive fatigue of a patient with multiple sclerosis—a concern implicitly raised by Fernández-Cañas et al. (Contribution 5) in their review of AR/MR interventions; or an exoskeleton that prioritizes biomechanical efficiency but overlooks the patient’s fear of falling, generating anxiety rather than confidence; or a monitoring system whose constant presence erodes the very privacy and autonomy it was meant to protect.

This tension is not hypothetical. It is visible in the abandonment of assistive technologies, in the frustration of users confronted with poorly adapted interfaces, and in the emotional disconnect that arises when technological systems fail to resonate with the person’s cognitive or cultural reality [4]. Designing technologies that truly serve the person requires more than technical precision—it demands a proactive ethical framework that places user needs and values at the center from the very outset [5,6].

A mature neurorehabilitation field must therefore embrace this dialectic: technology is powerful, but its power must be governed by ethics, sensitivity, and humility.

## 3. Neuroplasticity Is Not Only Biological—It Is Relational

A third insight emerges when we consider neuroplasticity. The studies in this Special Issue implicitly challenge the idea that neuroplasticity is a purely biological process. Instead, they show that neuroplasticity is contextual, relational, and experiential.

The brain reorganizes not only in response to stimuli, but in response to motivation, emotional safety, meaningful engagement, social connection, and the sense of being understood. Social factors, in particular, play a unique role in shaping recovery after neurological injury. Experimental and clinical evidence has shown that social isolation can increase stroke incidence and impair recovery, while social interaction enhances neurogenesis and reduces neuronal death [7]. More recent work on inter-brain plasticity has demonstrated that the coupling between brains during social interaction persists after the interaction ends and predicts the motivation to connect with others, suggesting that social bonds leave lasting neural traces [8]. The experience of rewards derived from positive social interactions encourages the sharing of perspectives and preferences that unite individuals, helping to buffer against the consequences of stress and isolation [9].

Rapolienė et al. (Contribution 3) explicitly frame their balneotherapy findings in these terms, arguing that stress reduction and improved autonomic regulation can support neuroplasticity, recovery processes, and overall quality of life. Technological interventions succeed when they create conditions that support these human factors. They fail when they ignore them.

Thus, neuroplasticity is not activated by technology alone. It is activated by the quality of the experience that technology enables. A VR system that overwhelms or confuses will not promote plasticity. A mixed-reality task that resonates with personal history or identity—as Franzò et al. (Contribution 4) emphasized when they note that occupational therapy is especially beneficial if focused on meaningful activities—can unlock motivation that no algorithm can generate.

This reframing invites a new paradigm: neuroplasticity is not only a biological opportunity—it is an ethical responsibility.

## 4. Dignity, Vulnerability, and the Ethics of Rehabilitation

Neurological disorders—whether it be stroke, multiple sclerosis, traumatic injury, or dementia—introduce vulnerability. And vulnerability demands ethical vigilance. Patients are not “users” of rehabilitation technologies. They are subjects of rights, with histories, fears, hopes, and identities. As we have recently proposed through a “Dignity-by-Design” framework in the context of assistive technologies, the institutional drive to deploy digital tools must be evaluated not only by their precision and efficiency, but by their alignment with the lived experience of vulnerable individuals [6]. This principle is particularly relevant when considering the immersive VR intervention for dementia by Latorre et al. (Contribution 1): beyond its physiological effects, what makes it ethically sound is its capacity to create a safe experiential space that respects the person’s emotional reality, rather than reducing them to a set of cognitive scores. Rehabilitation must therefore protect autonomy, emotional truthfulness, cultural resonance, and the right to be treated as a person rather than a problem to be solved.

This Special Issue shows that dignity is not an abstract concept. It is a practical design requirement. It is present when: interfaces adapt to cognitive load, tasks respect personal meaning—as with the patient-specific mixed-reality protocol of Franzò et al. (Contribution 4)—systems avoid overwhelming sensory input, monitoring does not become surveillance, and technology amplifies human connection rather than replacing it.

The ethical horizon of neurorehabilitation is clear: technology must serve the person, not the other way around.

## 5. Toward a More Human Neurorehabilitation

Taken together, the contributions of this Special Issue point toward a future where neurorehabilitation is technologically sophisticated, clinically rigorous, ethically grounded, and deeply human.

The challenge ahead is not simply to refine devices or optimize algorithms. It is to cultivate a rehabilitation ecosystem where innovation is guided by empathy [2], where neuroplasticity is understood as relational, and where dignity is treated as a measurable outcome rather than a philosophical aspiration [6].

If rehabilitation is to restore function, it must also restore humanity.

If technology is to empower, it must also listen.

If neurorehabilitation is to evolve, it must do so without losing sight of the person at its center.

This Special Issue invites us to measure success not only in gait speed or cognitive scores, but in the quiet victories: a patient who smiles during a VR session, a family member who sees recognition in a loved one’s eyes, and a moment of agency reclaimed from the grip of disease. Such a future is not only possible—it is already emerging.
